# Abdominal myoepithelial carcinoma: A rare abdominal wall entity of an uncommon tumor

**DOI:** 10.1016/j.ijscr.2022.107618

**Published:** 2022-09-09

**Authors:** Daania Shoaib, Saqib Raza Khan, Yasmin Abdul Rashid, Muhammad Nauman Zahir

**Affiliations:** a Aga Khan University Hospital, Karachi, Pakistan; b Dr. Ziauddin Hospital, Karachi, Pakistan

**Keywords:** WHO, World Health Organization, CT, Computed Tomography, IHC, immuno-histochemical, MDT, multidisciplinary tumor, WLE, Wide Local Excision, NGS, Next Generation Sequencing, ME, myoepithelial, EMA, Epithelial Membrane Antigen, FISH, Fluorescence in situ Hybridization, EWSR1, Ewing Sarcoma Breakpoint Region 1, EMC, Extra skeletal myxoid chondrosarcoma, Myoepithelial carcinoma, Abdominal malignancy, Extra-salivary

## Abstract

**Introduction and importance:**

Myoepithelial carcinomas are a diverse group of tumors exhibiting myoepithelial differentiation. There have been increasing reports of extra-salivary sites of origin for myoepithelial carcinomas such as soft tissues, bone and visceral areas. Due to this entity's rarity, definite diagnostic and treatment parameters are somewhat limited. We present the case of a myoepithelial carcinoma arising from the abdominal wall, a rare site of origin of an uncommon tumor.

**Case presentation:**

A 37-year-old gentleman presented to our institution in Oct 2018 with a recurrent abdominal mass for which he underwent wide local excision after completing the workup, which included systemic scans and relevant blood investigations. The histopathology report was consistent with malignant abdominal myoepithelial carcinoma. However, subsequent follow-up scans in May 2019 showed disease progression with the appearance of multiple lung metastases. After a detailed discussion, he was started on Pazopanib 800 mg orally once a day, on which he remained stable till May 2022. It was then when he experienced clinical disease progression confirmed on systemic scans, so he was offered palliative systemic chemotherapy.

**Clinical discussion:**

Abdominal malignant myoepithelial carcinomas are an infrequent entity. However, this case highlights its critical diagnostic markers and primary and recurrent abdominal myoepithelial carcinoma management.

**Conclusion:**

Abdominal myoepithelial carcinomas, although rare, are also under-recognized. Thus, keeping an index of high suspicion for these tumors and being armed with knowledge regarding the heterogeneity of its features would lead to better diagnostic awareness and documentation, paving the way for better evidence-based treatments.

## Highlights

• Extra-salivary myoepithelial carcinoma is an extremely rare entity, may present as an abdominal mass.
• In contrast to their salivary-gland counterparts, most of them are malignant to begin with.
• Limited data is available for their management, hence such patients should be enrolled in clinical trials whenever available.

## 1 Introduction

Myoepithelial tumors are a diverse group of tumors exhibiting myoepithelial differentiation [1]. Benign and malignant neoplasms of myoepithelial cells comprise a relatively rare entity, with the World Health Organization (WHO) having updated its classification of them as recently as 2013 and adding them to the category of tumors of uncertain differentiation [2].

Myoepithelial cells are found most commonly surrounding the ducts of various glands in the body. Thus, under their formative cells, these tumors are typically expected to be found in structures containing glandular or ductal tissues. Myoepithelial carcinoma, or malignant myoepithelioma, was first studied by Stromeyer et al. in 1975 [3]. These tumors show a wide range of cytological variations, including spindle, plasmacytoid, epithelioid, and clear cell type [4]. The relatively better-known site of origin of these rare tumors is the salivary glands, with the parotid gland leading the board [3]; however, even here, their incidence accounts for less than 1 % of all salivary gland tumors [3], [4].

Over the past decade, there have been increasing reports of extra-salivary sites of origin for myoepithelial carcinomas such as soft tissues, bone, and visceral areas [1], [5]. Soft tissue origins are highly uncommon. They tend to predominantly arise from the limb girdles & extremities, with other appendicular sites being scarce [5]. A large proportion of cases are seen in the pediatric population, with there being an equal propensity for both genders in adults [2], [5] and usually coming to light before the 4th decade of life [1], [2].

In contrast to their salivary gland counterparts, most soft tissue myoepithelial tumors are malignant, to begin with [1]. The argument for malignancy is based on the degree of cytologic atypia in contrast to the criterion of invasive growth in salivary gland sites [6]. Prompt differentiation of myoepithelial carcinoma has clinical value compared to its benign counterpart; they tend to have a higher rate of local recurrences and distant metastases [7]. Owing to the rarity of this entity, definite diagnostic and treatment parameters are somewhat limited, which can often render the management of myoepithelial carcinomas challenging [8].

Here we present the case of a myoepithelial carcinoma arising from the abdominal wall followed over a few years of treatment at our institution, to add to the sparse literature present for this particular site of origin.

## 2 Case presentation

A 37-year-old gentleman, married and resident of Karachi, Pakistan, presented in our institution in the fall of October 2018. He had a previous history of swelling in the right inguinal region, which first came to his notice in 2010. Since there were no accompanying symptoms with this solitary swelling, he sought no active treatment. However, by 2018, the node had continued to increase progressively in size. This complaint was investigated and managed at another healthcare facility outside our institution, where he underwent an excision of the mass in January 2018. En-bloc surgical resection of the tumor was performed via the right flank incision. Intra-operative findings revealed that the tumor mass involved the internal oblique and transversus abdominis, however; there was no evidence of distant disease. The tumor was excised en-bloc, and a healthy, negative tissue margin was achieved. The abdominal wall defect was closed using mesh, sutured, and the subcutaneous tissue and skin were closed in a standard protocol fashion. A histopathology sample was sent, and it was reported in our institution as a neoplastic lesion arranged in lobular configuration with lesional cells that are spindle in shape with moderate atypia and composed of cords and nests.

**Fig. 1:**
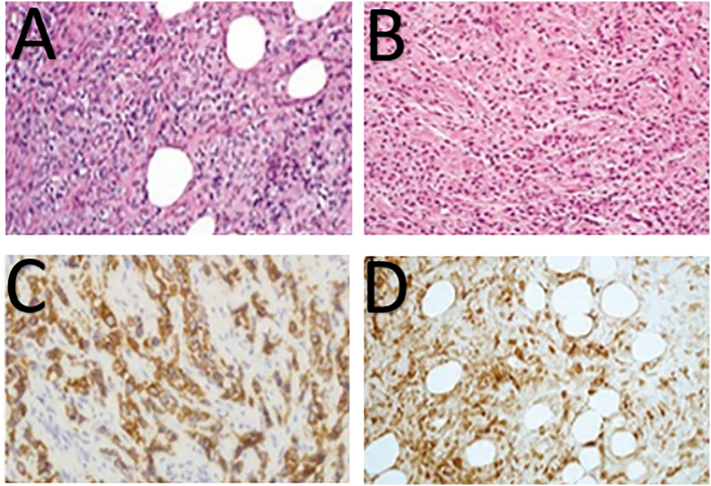
(A-D) Histopathological features of malignant myoepithelial carcinoma, composed of cords and nests, showing clusters of uniform cells with eosinophilic cytoplasm (A, B). Strong IHC expression of cytokeratin and showing cord like pattern (C) and strong IHC expression of S-100 protein (D).

Furthermore, the immunohistochemical (IHC) stains showed P63 positivity with Cytokeratin AE1/AE3 focal positive, S-100, and EMA patchy positive. These findings favor myoepithelial carcinoma of the abdominal wall, an infrequent entity (Fig. 1A-D). He remained stable from Jan till Oct 2018.

**Fig. 2:**
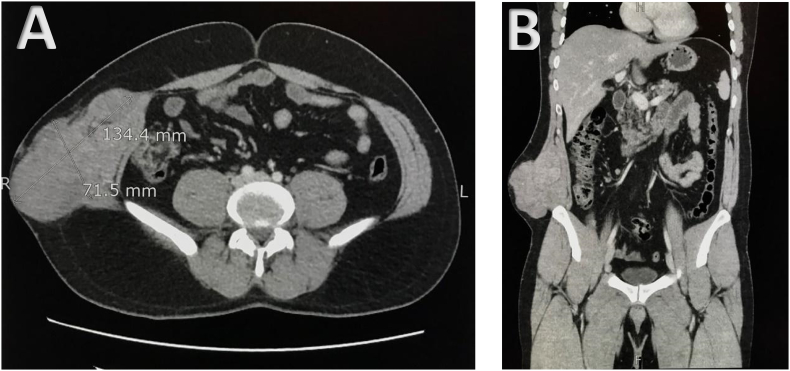
(A-B) Computed Tomography (CT) scan (October 2018) in axial (A) and coronal (B) views showing the primary mass arising from the right lateral abdominal wall.

He now presented with complaints of a rapid increase in the size of primary mass over the span of a few months. On examination, he was of average height and built, and all his vital parameters were within reference ranges. No lymphadenopathy was appreciable. The systemic examination revealed a right-sided previous surgical scar over the abdomen and a large, firm mass, approximately 10 × 10 cm in size, non-tender with no overlying skin changes and fixed to the underlying abdominal wall, extending from the right lower lumbar region to the inguinal area. The rest of the systemic examination was unremarkable. Relevant laboratory investigations were all within normal reference ranges as well. A Computed Tomography (CT) scan was carried out in October 2018 to restage the disease, which showed hypermetabolic soft tissue mass with a necrotic component of the right lateral abdominal wall – 134.4 × 71.5 mm (Fig. 2A-B). The remainder of the systemic scan did not show any distant metastasis. The case was discussed in our Multidisciplinary institutional tumor (MDT) board meeting, and the recommendation was to undertake surgical resection of the tumor. Subsequently, he underwent Wide Local Excision (WLE) of the right abdominal wall lesion with an anterolateral thigh flap in November 2018. The surgical specimen was consistent with the previously known pathology. Post resection of the tumor, his clinical condition continued to improve; however, he became lost to follow-up and later presented in May 2019 with a repeat CT scan which showed significant disease progression with local recurrence of the disease and lymphadenopathy as well as interval appearance of multiple soft tissue nodules in bilateral lung fields.

Despite the lack of a definitive and standard chemotherapeutic regimen for recurrent metastatic myoepithelial carcinoma, options for platinum and taxane-based regimens were offered. In addition, a detailed discussion in the context of treatment and prognosis was done with the patient, after which he was started on Pazopanib. This multiple kinase inhibitor limits tumor growth by targeting angiogenesis with a dose of 800 mg orally per day.

Next Generation Sequencing (NGS) was sent on the pathological specimens to identify any targetable mutation, results of which did not return any mutation that could serve as a therapy target. On subsequent follow-up scans, he remained clinically stable with improvement in his disease process; hence, he continued on Pazopanib.

**Fig. 3:**
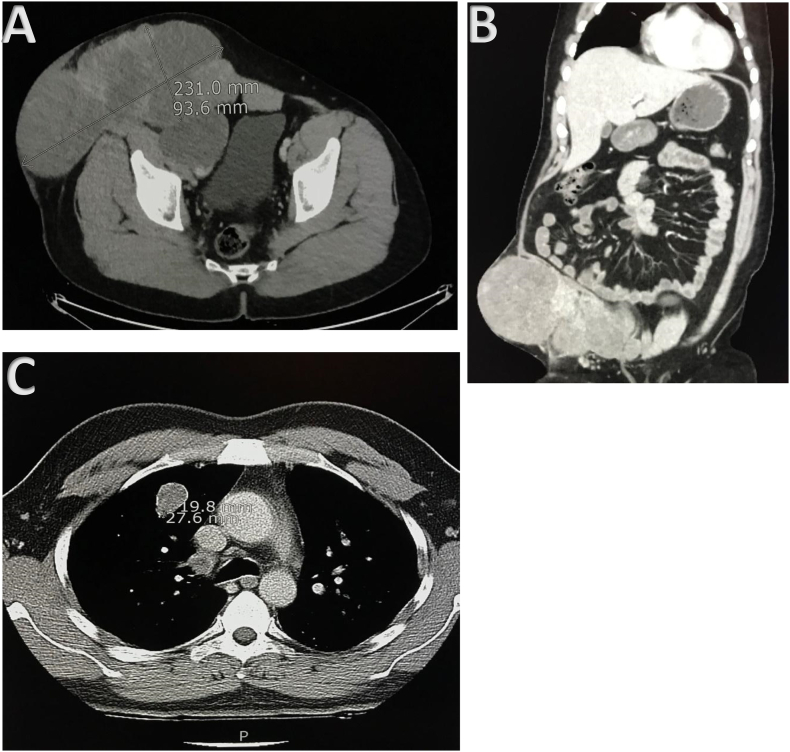
(A-C) CT scans from the most recent presentation (May 2022) in axial (A) and coronal (B) views, respectively. Gross progression of the disease can be seen with regional lymphadenopathy and a conglomerate mass. Also, CT scans in axial view (C) demonstrating the progression of metastatic pulmonary deposits, both in size and number.

He most recently presented to the clinic in May 2022 with complaints of abdominal discomfort. Examination revealed a large palpable inguinal mass of approximately 10 × 15 cm. A repeat imaging scan was performed, which showed significant disease progression (Fig. 3A-C). As a result, his case was re-discussed in the MDT meeting, and he was offered palliative systemic chemotherapy with Cyclophosphamide (500 mg/m^2^ intravenously), Doxorubicin (50 mg/m^2^ intravenously) and Cisplatin (50 mg/m^2^ intravenously) on day 01 of each 28-day cycle.

All the work has been reported in line with the SCARE 2020 criteria [9].

## 3 Discussion

Myoepithelial (ME) carcinoma, also known as malignant myoepithelioma, is an entity within a group of neoplasms classified as tumors of uncertain differentiation. These tumors, which are primary salivary gland tumors, although rare, are characterized well in literature. In contrast, data regarding primary soft tissue myoepithelial tumors is scarce. Burke et al. were the first to report a retroperitoneal soft tissue ME tumor in 1995 [10]. Over the past decade, these tumors have started to gain more recognition owing to advanced diagnostic techniques. Most of the ME tumors that arise from soft tissues are malignant. An apparent gender preference has yet to be reported in them. Age is not a barrier to diagnosis either, with cases ranging from 02 to 83 years, peaking in the 4th to 5th decade of life. When diagnosed in children, which is the case roughly 20 % of the time, these tumors prove to be malignant in around two-thirds [5], [11].

ME carcinomas usually are slow growing with little to no symptoms observed. However, when reported, there may be a swelling noticed to be growing over time, pain or paresthesia, or other symptoms linked to metastatic sites. Studies show that symptoms can range from 2 weeks to several years [12], [13]. The most common regions from where ME carcinomas arise are the extremities. When Lee et al. reviewed 120 cases of ME carcinomas, they found that nearly 70 % originated from upper and lower limbs (40 % & 31 %, respectively), with around 10 % arising from the trunk. Other sites may be the viscera. Hornick and Fletcher also reported similar figures in their investigation [14], [15].

Myoepithelial soft tissue tumors are considered to be of uncertain histogenesis since they lack any regular cellular counterpart, with normal ME cells not found at these sites [5], [12]. Therefore, these tumors are relatively heterogeneous in terms of cytology and architecture. The most commonly observed cell types are epithelioid, plasmacytoid (hyaline), spindle and clear cell. Typically, the epithelioid cells are paired up with one or more of the other cell types [13], [16], as evident in our case being the epithelioid-spindle cell type. These cells are then arranged in various formations, reticular patterns most commonly seen in soft tissue tumors. Trabecular, nested or solid patterns have associations with other subtypes [16].

Principles of malignancy concerning soft tissue ME carcinoma primarily focus on the degree of cytological atypia, usually moderate to severe, with the present nuclear pleomorphism. High mitotic counts and tumor necrosis are also common. This feature contrasts with ME carcinomas of the salivary gland, where the most significant feature of malignancy is infiltration and invasive growth [1], [5]. In their comprehensive study, Hornick and Fletcher emphasized that in the setting of severe cytological atypia, there was a statistically significant difference in metastasis and recurrence, elucidating the link to aggressive behavior [4], [8], [15].

Histological features may often be overlapping and thus unable to definitely diagnose ME carcinomas. Since the advent of immunohistochemical staining, accurate diagnosis has resulted in a significant breakthrough. When myoepithelial cells undergo neoplastic transformations, specific proteins are expressed, which can be picked up via immunohistochemistry [16]. Most ME neoplasms co-express both epithelial and myogenic markers. As such, panels which include pan-cytokeratins &/or Epithelial Membrane Antigen (EMA) along with S-100 &/or GFAP can accurately identify myoepithelial carcinomas [5], [11], [16]. Other markers which may be identified are SMA, Calponin and p63. Frequently, myogenic markers may be lost, but this does not rule out ME tumors, as it is well established that neoplastic myoepithelial cells often lose expression of myogenic markers [4].

Despite being essential to diagnosis, the problem with immunohistochemical markers was that the very same was also positive in the myoepithelial neoplasm of the salivary glands. Thus efforts were focused on identifying any genetic markers using Fluorescence in situ Hybridization (FISH). Pleomorphic adenomas of the salivary gland have characteristic fusion oncogenes, namely Pleomorphic Adenoma Gene 1 (PLAG1). Interestingly, this alteration was not detected in studied soft tissue myoepithelial tumors [16]. It was Antonescu et al. who had a breakthrough in 2010. While investigating a larger population of ME tumor cases, they identified that nearly 45 % were found to contain Ewing Sarcoma Breakpoint Region 1 (EWSR1) alterations, out of which almost two-thirds were of soft tissue origin [17]. Since then, multiple studies have corroborated this, with literature suggesting that nearly 45 % of all soft tissue ME carcinomas have this gene rearrangement [1], [5], [11], [12]. EWSR1 is located on chromosome band 22q12 and encodes a transactivator. This rearrangement is also seen in other neoplasms such as Ewing sarcoma, Desmoplastic small round cell tumor and Extraskeletal myxoid chondrosarcoma [8]. Myoepithelial carcinomas of soft tissue with this particular rearrangement have been shown to follow a much more aggressive course [1], [11].

Owing to the morphological similarity to other neoplasms, a combination of histological, immunohistochemical and genetic testing helps to thin out the differential diagnosis. For instance, Extra Skeletal myxoid chondrosarcoma (EMC) has rhabdoid features which mimic the plasmacytoid morphology of myoepithelial carcinomas. However, EMC has a lobular pattern & lacks expression of GFAP, epithelial & myogenic markers. Characteristic features of other common differential diagnoses are; epithelioid sarcoma, with S-100 expression being rare and negative GFAP, sclerosing epithelioid fibrosarcoma with negative keratin and S-100. The absence of S-100 and GFAP expression and the lack of epithelial markers also exclude metastatic carcinoma or melanoma, respectively [8], [12].

Metastasis is commonly seen with these tumors. Nearly half of all diagnosed cases risk metastatic disease [1], [5], [18]. The most common site of metastasis is the lung, followed by lymph nodes, soft tissues and bone [1]. An interesting observation is that this pattern of spread encompasses features of both carcinomas (lymph nodes) and sarcomas (lung) [13].

Due to the challenges in forming an accurate diagnosis of these tumors and their rarity, guidelines for optimal management remain poorly defined. The current foundation of management, especially for localized disease, remains complete surgical resection with clear margins & this may be followed by postoperative radiation [1], [18]. With clear margins, disease-free survival can range from 14 to 85 months [16]. As for systemic chemotherapy in the metastatic setting, there is too little data to formulate a regimen and test its outcomes. Within the available literature, variable results have been seen in response to chemotherapy. For instance, in an investigation by Chamberlain, the median progression-free survival observed following first-line chemotherapy was around 9.3 months [1].

In stark contrast, in 2006, Noronha et al. published a fascinating report. It elaborated on a case of metastatic soft tissue ME carcinoma of the vulva treated with a chemotherapeutic regimen of Carboplatin & Paclitaxel. Following 3 cycles, there was a complete radiological response seen. This was consolidated with three more cycles, and progression-free survival was seen up to 42 months post-treatment [19].

Despite utilizing the current standard of treatment, these tumors have an aggressive tendency and, as such, recur commonly, with multiple studies suggesting a recurrence rate between 35 and 45 % [1], [5], [18]. This figure makes long-term follow-up and surveillance an essential tool in management.

Due to the scarcity of data and the significant morbidity these tumors can cause, clinicians should encourage enrollment in clinical trials. Alongside these steps, further research into the ESWR1 rearrangements and other genetic markers would better understand what makes these tumors tick and develop efficient, targeted therapies.

## 4 Conclusion

Abdominal myoepithelial carcinomas, although rare, are also under-recognized. Thus, keeping an index of high suspicion for these tumors and being armed with knowledge regarding the heterogeneity of its features would lead to better diagnostic awareness and documentation, paving the way for better evidence-based treatments.

## Funding

We have NO funding available.

## Ethical approval

ERC approval for publication of this case report was taken from Aga Khan University Hospital.

## Consent

Written informed consent was taken from the patient for publication of this case report.

## Registration of research studies

Not applicable.

## Guarantor

Corresponding author is the guarantor of this case report.

## CRediT authorship contribution statement

**Daania Shoaib:** Writing – original draft. **Saqib Raza Khan:** Writing – review & editing. **Yasmin Abdul Rashid:** Conceptualization. **Muhammad Nauman Zahir:** Supervision.

## Declaration of competing interest

Authors have no conflict of interest.
